# The Arabic Version Validation of the Social Worries Questionnaire for Preadolescent Children

**DOI:** 10.3390/children12080994

**Published:** 2025-07-29

**Authors:** Asma Saraireh, Basim Aldahadha

**Affiliations:** 1 Department of Psychology, Mutah University, Mutah 61710, Jordan; asmasar@mutah.edu.jo; 2 Department of Counseling and Special Education, Mutah University, Mutah 61710, Jordan

**Keywords:** social worry, preadolescence, validity, reliability, assessment

## Abstract

Background/Objectives: Social worry may appear in preadolescence, causing it to persist in the future, such that adolescents become more avoidant of situations in which they are evaluated by others. Many previous studies have shown that approximately 4% of preadolescent children suffer from high levels of social worry, which may lead to other problems in the future. It is important to take necessary precautions to explore this worry and take the necessary measures to address it. This study aimed to do so by extracting the psychometric properties of the Social Worries Questionnaire (SWQ) for preadolescence in Arab countries. Methods: The sample consisted of 269 children aged 8–12 years. The SWQ, Spence Child Anxiety Scale (SCAS), Child Abuse Self-Reporting Scale (CASRS-12), and Arabic version of the Children’s Depression Inventory (AVCDI) were administered via various social media. Results: Confirmatory factor analysis was used, and the one-factor model showed that the scale consists of 14 items. The results also indicated evidence of discriminant validity, and that the internal consistency was acceptable. For reliability, the test–retest results showed that the value was appropriate. Conclusions: This scale could be valuable in detecting worry in preadolescent children and providing them with therapeutic and psychological services.

## 1. Background

Social worry refers to the fear of people evaluating them, especially in social interaction situations, school presentations, and academic achievements [1,2]. Low levels of social anxiety can be considered healthy and positive adaptations, but cases in which worry appears to be exaggerated indicate a state of discomfort and affects academic efficiency, daily achievement, and interaction with others [3,4,5].

This problem includes symptoms that appear in generalized worry disorders in terms of physical symptoms (e.g., sweating, facial flushing, dry throat, and stuttering), as well as cognitive symptoms that indicate anticipation of catastrophic events, destructive thinking, and anticipation of evil due to fear of others’ evaluations and judgments of their achievements in general, and behavioral symptoms including avoidance, escape, and engaging in behaviors that the person believes are safer in academic and social situations [6,7]. Children sometimes express worry toward social situations through crying, screaming, vandalism, distrust of others, and perhaps failure to achieve, struggling, or stopping [5,8].

Social worry generally begins at the age of 10 years, depending on several factors, the most important of which are cognitive development and the extent to which the child acquires skills that help interact with others and help them understand their point of view, which would help build social relationships and perform schoolwork better and with distinctive success [9]. With respect to social worry, studies conducted by Solmi et al. [10] reported that half of the sample members had symptoms of social worry at the age of 13 years or earlier and that 25% of these patients had symptoms at the age of 7 years or earlier.

On the other hand, research conducted on the mental development of these problems indicates that they are likely to remain and continue beyond childhood and may increase further during preadolescence, especially in children over 10 years of age. Canals et al. [11] reported that approximately 4% of Spanish children aged 9–12 years have symptoms that indicate high levels of worry, especially in females. Orgilés et al. [12] noted that 4.6% of children and adolescents aged 8–17 years presented high levels of social worry and that what mostly explains these problems is their significant association with one or more other problems, such as depression, substance use, internet misuse, addiction to electronic games, and academic difficulties.

The assessment of the SWQ in children and adolescents has been followed and achieved through interviews with both children and their parents and through observations, whether in therapeutic situations with the presence of parents or through observation of children in natural situations when they are of an appropriate age and can be administered with scales and questionnaires [7]. The use of self-report scales by children is a very useful method and is widely used, as are scales by which parents respond to their children’s behaviors. However, discrepancies are most evident in the diagnostic reports of social worry symptoms between parent reports and children’s reports themselves, and there are differences between these different sources of worry [13].

As stated by De Los Reyes et al. [14], there is a low-to-moderate correlation between self-report measures and the reports of other people who act on their behalf to measure certain problems, such as social worry. The minimum age at which it can be used to judge honestly is controversial according to several psychological characteristics and factors. In any case, the age of 7 years is considered suitable for providing stable and reliable results and self-reports [15], especially if these items are simple and include limited options of only two to three options [16]. For this reason, self-report measures may be preferable for assessing children’s entry problems, and questionnaires are considered stable, valid, and appropriate for preadolescents.

As mentioned by Tulbure et al. [17] (2012), most Arabic tools used to measure social anxiety are directed at adolescents, and there are no Arabic tools or self-report scales targeting ages ranging from 8 to12 years, other than those used in this study. We recommend the use and activation of these tools, which are directed toward Arabic-speaking children [17,18], whereas many scales have focused on social worry in preadolescent children in other cultures and languages [19,20,21,22].

A review of the SWQ revealed very strong psychometric properties for both the Spanish-standardized version [8] and the original English version [23,24]. A review of these instruments and situations revealed that they focus on meeting new people, taking tests, expressing disagreement, expressing personal preferences, talking on the telephone, going to parties, speaking in front of others in class, or going to the school cafeteria [19,20,21,25]. There is also what is called videoconference anxiety, a new type of worry that needs further research and may be of interest to future scientific research and has not been addressed in previous scales [8,26].

In research and clinical settings, it is important to know which specific situations generalize anxiety due to possible judgments from others and to what extent these judgments can extend to generalizing worry and prejudice from others, as is the case for generalizing problems related to anxiety [6,8]. Some scales have focused on concrete measures of the response to worry in children rather than situations that elicit worry. Conversely, these instruments focus on the same situations and contain many items. In contrast, several studies have demonstrated the effectiveness of using short scales [27,28]. Therefore, a small number of items can be particularly useful to assess situations that can generalize social worry. To date, there is no reliable Arabic tool for this purpose. In addition, the available scales cover the ages of adolescence and beyond, and none of these are specifically directed at preadolescent children.

One measure that can bridge this gap is the SWQ developed by Spence [24], which consists of 13 items that measure worry in specific social situations that may involve negative evaluations from others. These situations included talking on the telephone or eating in public places. They were selected from the literature, scientific research, and standardized interviews with children with social worry. The original version of this measure was administered to 386 Australian children and adolescents.

The results of a factor analysis of the single factor showed good internal consistency (α = 0.84) and construct validity for the SWQ. The results revealed that the means did not differ significantly according to the gender or social status of the children [23]. This scale has been used in a wide range of studies and has been very effective in assessing social worry disorders in children and verifying the effectiveness of therapeutic methods in dealing with social fears [29] and autism spectrum disorders [30], as is also the case in the research relationship between them [31].

This research aimed to adapt and standardize the SWQ and extract its psychometric properties for children in the Arab environment, noting that the original version of this instrument was applied to a sample of children aged between 8 and 17 years [23]. In this study, this scale was standardized for preadolescent children, and the sample was 8–12 years of age [8], with the aim of early detection of social worry in Arabic-speaking children and taking the necessary precautions to protect children and rapid intervention for possible psychological treatment.

## 2. Methods

### 2.1. Participants

The participants included 269 urban children, 55.76% boys, (n = 150) aged from 8 to 12 years, with a mean age of 9.60 years (SD = 1.40). Table 1 shows the demographic characteristics of children and their parents. The table indicates that the percentage of children living with their parents is 78.81% (n = 212), and the percentage of those holding a bachelor’s degree is 29.37% (n = 79). It is clear from Table 1 that most of the children 27.14% were 12 years old (n = 73), most of their fathers held a diploma or less 47.21% (n = 127) and the percentage of divorced fathers was 10.41% (n = 28). The socioeconomic level reached a moderate level at 48.33% (n = 130), the middle child had the largest percentage 46.46% (n = 125), and the mean family size ranging from 4 to 6 had the largest percentage at 53.53% (n = 144). On the other hand, the percentage of Jordanian children is 95% (n = 255), while the remainder are Iraqi and Syrian nationalities. Finally, the total number of children who received psychological treatment for one or more sessions for any reason during the one-year period of study implementation was 51.5% (n = 139).

### 2.2. Instruments

First, the guardian responsible for the child or the legal authority recorded the data and demographic characteristics of the participating children in terms of the sex, age, and psychological treatment they received. Subsequently, the children, at the behest of their parents, completed the self-assessment scales for the following tools.

*The Social Worries Questionnaire* (SWQ) [23,24] measures worry and avoidance during the past four weeks toward various situations of achievement, social interaction, and exposure to others. This scale consists of 14 items answered on a three-category scale: 0 = not true, 1 = sometimes, and 2 = often true. Accordingly, the highest score on this scale indicates a greater degree of generalization to avoid situations that arouse fear and social worry. The total score on this scale ranges from zero as minimum to 28 maximums. This scale was standardized by the International Testing Committee and the guidelines followed by this institution. Two researchers who were fluent in English and Arabic translated the scale into Arabic and verified that the items convey the same meaning. The items were modified to suit the children’s understanding and to be easy and simple, not complex, and frequently used words. Subsequently, the scale was translated from Arabic to English via reverse translation to verify the extent of compatibility and similarity between the original English version and the reverse translation of the English version. Finally, the scale was presented to another group of arbitrators, 10 faculty members from various educational and psychological specializations, to judge the soundness of the language, the degree of its belonging to the measurement, and the degree of the child’s ability to understand it. In this round, some words were replaced, others were deleted, and others were added. The modifications were simple but at the same time, important and useful. The results revealed an acceptable internal consistency (α = 0.82).

The SCAS [18,32] consists of 8 items answered on a 4-point scale from 0 = never to 3 = always, with a higher score indicating a higher level of worry. The total score of this scale ranges from 0 as a minimum to 24 as a maximum. The authors of this scale translated and standardized it in the Arab environment for children aged 8–12 years through the factorial construction of a single dimension. The results revealed an acceptable internal consistency (α = 0.84).

*Child Abuse Self-Report Scale* (CASRS-12) [33] consists of 12 items in Arabic. The scale consists of three categories: never = 0, sometimes = 1, always = 2, where a higher score indicates a higher level of exposure to abuse. The total score for this scale ranges from zero as minimum to 24 maximums. The scale items are distributed across four factors: psychological abuse (PSA) (e.g., My parents treat me with disrespect), physical abuse (PHA) (e.g., I testify that other members of my family are being beaten), sexual abuse (SEA) (e.g., An adult makes me look at or touch his/her private parts), and neglect (NEA) (e.g., I spend a restful life). Each factor contained four items. The scale was presented to a group of experts and clinical specialists in the field of child health in both Lebanon and Tunisia, including the authors of this study. Similar items that contained the same words were excluded to reduce similarity. The correlations between the items were very high (r = 0.90). Notably, the scale was applied to a sample of 404 children with a mean age of 16 years. The results revealed high internal consistency, ranging from α = 0.87 to α = 0.93, between the subdimensions.

*The Arabic version of the Children’s Depression Inventory* (AVCDI) [34] consists of 28 items that measure children’s depressive symptoms. These items were answered on a three-category scale: zero means no symptoms, one means moderate symptoms, and two means severe symptoms, with the highest score indicating very severe depressive symptoms. The total score for this scale ranges from zero as a minimum to 56 maximums. The participants chose items that described their psychological state and the emotions they have been experiencing during the past two weeks. This scale took 15 min to complete. The scale was applied to a sample of 860 children aged between 8 and 14 years with a mean age of 9.28 years (*SD* = 7.30) from a sample of school children in Egypt. This scale has appropriate psychometric properties. The test–retest validity was conducted, and the correlation coefficient was (*r* = 0.76) 21 days after the first application. A Cronbach’s alpha test was also conducted, and the internal consistency results revealed that the value of *F* was α = 0.79 for males, whereas it was *α* = 0.84 for females. Many Arab studies have been conducted on this scale to verify the validity of the construct, and it was found that the correlation coefficient of this scale with the Beck Depression Inventory was (r = 0.87).

### 2.3. Procedure

The procedures were implemented by communicating with the study individuals through various social media networks, WhatsApp, Facebook, Twitter, Instagram, and e-mails, for a period of 10 days to obtain sufficient respondents. The link to the tools was sent to the people who had the largest possible number of followers. Each person was also asked to resend the link to the emails and other social media networks they had as part of recruiting via snowball sampling. These tools are organized according to the Google Drive program, so that the response is received and unloaded electronically. Notably, the tools were restricted and could not be sent except after all information was filled out without loss. On the other hand, these tools were sent to parents, and they were asked to provide information concerning them. They were also asked to have their children (aged from 8 to 12 years, both males and females) respond to these tools under their supervision. Notably, these tools can be used by children who are seven years old and above. They are parallel to the paper copies and can be an alternative and equivalent to them [35]. The first part of the social demographic information was included, and then they were asked to provide advice and supervise their children to complete the tools. Responses were received anonymously, and after eight weeks, the tools were sent back to the same channels to assess the stability of the responses over time, noting that none of the participants had received any financial support. Finally, these tools were filled out freely, and the first sentence on the scale was “If you do not want to participate, please do not answer and ignore the message”. Notably, many messages were sent, through followers of social media networks, estimated to be tens of thousands. However, the response was equal to the number of study individuals. The diagonal weight least squares (DWLS) estimator was used.

### 2.4. Statistical Analysis

Confirmatory factor analysis was performed using the diagonal weight least square (DWLS) estimator equation because a normal distribution was not achieved, which is suitable for the conditions of this study, as there are no assumptions about the distribution, and it is suitable for ordinal data [36] (Rhemtulla et al., 2012). Model fit indices were considered with the following criteria: RMSEA < 0.06; SRMR < 0.08 (or 0.09 in combination with RMSEA < 0.06); and CFI and GFI > 0.95 [37,38]. The factors of saturation, Cronbach’s alpha, residuals, ordinal alpha coefficient, internal consistency, and composite reliability were also analyzed based on the criteria of George and Mallery [39], which indicated an acceptable score of α > 0.70, a good score of α > 0.80, and an excellent score of α > 0.90. Convergent validity was tested with the minimum acceptable value of 0.50, whereas the mean variance extracted could be accepted if the composite reliability was greater than 0.60 [40,41].

Analysis of means, standard deviations and the Superman discrimination coefficient was conducted for each item and for the total score of the SAPC with the interpretation of Cohen’s criteria [42] (1988), which states that a weak relationship is (ρ > 0.10), whereas a moderate relationship is determined by the score (ρ > 0.50), and a strong relationship is (ρ > 0.30). Analysis of variance ANOVA of the variance model and the chi-square test (χ^2^) were also used according to the sex variable and the confidence level for the Mann-Whitney test, 95%. Version 23.0 SPSS was used for all statistical analyses.

## 3. Results

### 3.1. Descriptive Analysis

Descriptive analyses were conducted for each item within the range of 0–2, for which the study sample individuals answered on SWQ (Table 2). A mean of 0.80 or less for the items was considered a positive indicator of the symmetry of the data. The results showed that the symmetry index for all items fell within this category. In contrast, the item test Spearman’s correlations were conducted, and the results showed that these correlations were very high and excellent (except for some items 4 and 9, which were close to 0.50). This indicates good values and discrimination between the items, which can be relied upon to give them more confidence. The mean total score on the study tool was 8.46 (*SD* = 5.62).

### 3.2. Validity

The fit of the single-factor model was tested in the original version [23] and Spanish version [8] of the SWQ for children via CFA. Table 3 presents the constructed models. In Model 1, all items of the SWQ for children were considered to improve the fit of indicators. Model 2 was created alongside Model 1, in which the correlations of the residuals for items 10 and 12 in Model 1 were combined. Both items included a joint assessment of others confronting them, and the fear of sitting with people and crowded or closed places. Therefore, these two items share the same source of error.

The second model supported the fit indices, which were acceptable along with the parameters and integrations considered and was considered critical and final. All factors’ loading exceeded 0.50, except for items 4 and 9 which were greater than 0.42. The discriminant validity of the SAPC was assessed using other scales that focused on behavioral and emotional problems. Table 4 shows strong correlations over 0.50 of the SAPC scale with the AVCD inventory and the SCAS. Moderate correlations (>0.30) were associated with the SAPC, the CASR scale and all its factors. Finally, to test convergent validity, the mean variance (AVE) was calculated, obtaining a value of 0.43, which is lower than the proposed value of 0.50. The interpretation of this value, along with composite reliability, will be explained thereafter.

### 3.3. Reliability

**Figure 1:**
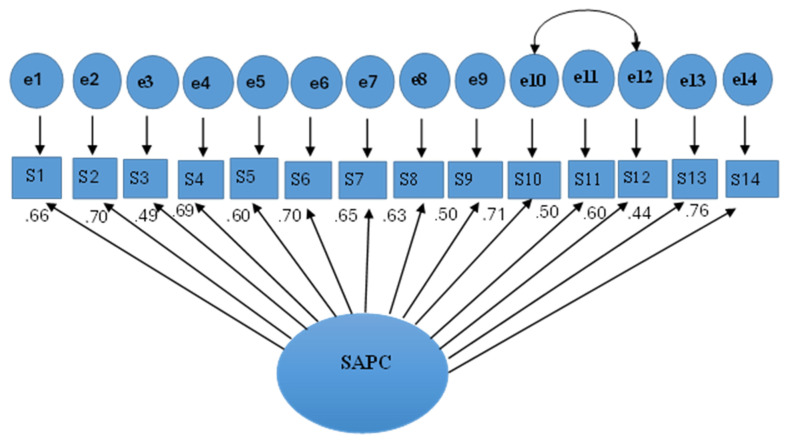
CFA model for the SAPC scale, including standard loading and standardized errors, n = 269.

The internal uniformity reliability of the scale was calculated using Cronbach’s alpha (α = 0.85) and the ordinal alpha (α = 0.82). The two results exceeded 0.80, which means that there is good internal consistency. On the other hand, the results of the composite reliability test revealed that its value was also 0.87, which indicates that there is a good percentage of common variance between the items. Finally, the results of the temporal stability of the SAPC were analyzed, as a subsample of 65 children (24.16%) was selected from the total sample. The results revealed that there were no statistically significant differences between the subsample and the rest of the original sample with respect to the total score on the SAPC scale (Mann-Whitney *U* = 3820; *p* = 0.203), age (Mann-Whitney *U* = 3958; *p* = 0.195), or sex (*p* = 0.274 (1, *N* = 269). The ICC was 0.80 [95% *CI* (59, 0.88), *F* = 4.39, *p* < 0.001]. Accordingly, these results confirm that there is a moderate to good degree of test-retest reliability (Figure 1).

## 4. Discussion

This study aimed to standardize the Arabic version of the social worry questionnaire for children aged 8–12 years. The Spence [23] scale was translated from English to Arabic. The Spanish version of the Amorós-Reche et al. [8] scale was used to add item 14, which included talking in a video call. The results revealed that it had a high and significant loading level. This study also aimed to extract psychometric properties in terms of validity and reliability on a sample of children from Jordan, Syrian, and Iraqi nationalities whose mother language is Arabic. The results showed that the items are clearly distinguished from each other and that there is a clear and significant contribution to the construction of social worries, which is expressed through various worrying situations through which others can evaluate the child, and which causes feelings of worry and avoidance. The correlation coefficients for the items were high and statistically significant and were better than those of the original and Spanish versions. In contrast, the model test was applied to verify the CFA and extent to which there was only one factor structure. The results showed that the loading was very similar to the original version [23,24] and the Spanish version [8] and that CFA had favorable fit indices.

To test the validity of concurrency, the results showed that there is a medium to strong relationship between the main study tool SAPC and the scales used, as there is a strong relationship between the study tool SAPC and both the AVCD inventory and the SCAS. While the relationship between the SAPC and CASR scales for the total score and its subdimensions was moderate, this result is consistent with previous research [8,43,44]. The strong and medium relationship can be explained by the existence of a common relationship that brings together the components of these tools and that all the items share in diagnosing situations of avoidance, threat, and fear of confronting and meeting others. These tools also share mutual diagnosis and negative impact, positive effectiveness, or behavioral control [45,46]; thus, this result enhances the reliability of this tool in detecting symptoms of social worry in preadolescent children. However, the relationships between the SAPC scale and the CASR scale and its various dimensions were moderate and not weak, which indicates the existence of a correlation that also explains the strength of the study tool. This is additional evidence of the strength of the items and discriminant validity between the various scales. This result can be interpreted as abuse related to harmful and immoral events, and at the same time, it somewhat expresses future worry, which explains the existence of a moderate relationship. From the point of view of all interpretations, there is clear evidence of correlation and discriminant validity between the SAPC scale and all scales. As a result, the Arabic version of the SAPC scale can be considered an appropriate scale for Arab and Jordanian environments, given the results that showed reliable psychometric properties with statistically significant results, including the original version and the 14 items that were added to the Arabic version.

Construct validity, represented by CFA, showed a strong-to-moderate relationship with the other scales. However, the AVE value was not within the previously determined range, which was higher than 0.50. In any case, the AVE value may have affected, in one way or another, the discriminant validity by reducing the level of variance between the items, perhaps because of the need to increase the number of items. This result is consistent with that of the Spanish version [8]. The positive results shown by the reliability values support the degree of reliability of this scale, which indicates that the single factorial construct items are suitable for this tool because of the presence of sufficient variance between the items. Considering this, we can say that there is additional evidence of reliability that appears through good internal validity, which is also largely consistent with the original version, which was α = 0.84.

On the other hand, the results of temporal stability revealed that there was stability ranging from medium to good, which explains why the age of the children was limited and why there was no follow-up of worry cases across the stages of adolescence, which provided specific information confined to a specific age group. Canals et al. [11] reported that 38.5% of young people are diagnosed with social worry. These previous studies explain how this problem is age-related and can continue beyond the age of 12. The results of this study are limited by several restrictions on the conditions for applying the study tools. Despite providing parents with sufficient instructions and verifying the possibility of understanding the questionnaire items and tools used and that the child reads these items under the supervision of the parents, there are some limitations that appear due to the difficulty of reading for some, which is a very small percentage of students, and it was addressed through the supervision of the parents and their assistance to their children in applying the tools. A second limitation is that the sample cannot represent all children in the Arab world; the results are more suitable for Jordan. And, we should not forget that there are large cultural differences within the Arab world. At the same time, these limitations may pave the way for future studies on SAPC.

## 5. Conclusions

This study concludes by presenting a standardized version of the SAPC scale with good psychometric properties for Arab children, in general, and Jordanians in particular, with the aim of detecting symptoms of social worry before adolescence, as social worry symptoms have an impact on a child’s future life. Notably, this study highlights the importance of the need for early detection of psychological problems for children, which is supported by UNICEF [47,48]. Moreover, the Arabic version of this scale can perform this task, as it will contribute to bridging the gap in the assessment of the psychological problems experienced by children, especially those who suffer from various abuses, which are evident through avoidance, fear, and worry from others. This Arabic version, translated from the original version [23], as is the case with the Spanish version [8], adds an additional item represented by worry through video calls, which provides more credibility. Notably, this tool contributes to assessing different situations for generalizing social worry, which is evident through avoidance, and is an important instrument for exploring other forms of psychological problems and different situations of worry. Moreover, there is no scale that measures worry in this age group. Finally, this version of the SAPC scale will open the door to conducting more studies and employing cognitive behavioral therapy to reduce social worry problems in preadolescent children by using the total score of the scale and applying various treatment methods in different categories of children.

## Figures and Tables

**Table 1:** Demographic characteristics of the participants.

Characteristics	% (n)
Child’s age	
8	14.50% (39)
9	19.70% (53)
10	20.44% (55)
11	18.22% (49)
12	27.14% (73)
The highest qualification of both parents or one of them	
Diploma and below	47.21% (127)
Bachelor	29.37% (79)
Master and above	23.42% (63)
Parent’s marital status	
Married and living together	78.81% (212)
Divorced or separated	10.41% (28)
Single	10.78% (29)
Socioeconomic status	
Low	25.28% (68)
Medium	48.33% (130)
High	26.39% (71)
Order of birth	
First	31.23% (84)
Mid	46.46% (125)
last	22.31% (60)
Family size	
3 and below	24.16% (65)
4–6	53.53% (144)
7 and above	22.31% (60)

**Table 2:** Descriptive analysis of SAP items.

	Items	Items (Arabic)	M (SD)	*ρi-t*
1.	Going to parties	الذهاب إلى الحفلات	0.40 (0.78)	0.55
2.	Using the telephone	استخدام الهاتف	0.48 (0.64)	0.53
3.	Meeting new people	التعرف على أشخاص جدد	0.52 (0.82)	0.58
4.	Presenting work to the class	تقديم العروض في الصف	0.48 (0.50)	0.44
5.	Attending clubs or sports activities	حضور النوادي أو الأنشطة الرياضية	0.75 (0.69)	0.58
6.	Asking a group of kids if I can join in	سؤال مجموعة من الأطفال عما إذا كان بإمكاني الانضمام	0.59 (0.77)	0.64
7.	Talking in front of a group of adults	التحدث أمام مجموعة من البالغين	0.73 (0.82)	0.50
8.	Going shopping alone	الذهاب للتسوق بمفردي	0.76 (0.89)	0.60
9.	Standing up for myself with other kids	الدفاع عن نفسي مع الأطفال الآخرين	0.80 (0.74)	0.46
10.	Entering a room full of people	دخول غرفة مكتظة بالناس	0.74 (0.63)	0.53
11.	Using public toilets or bathrooms	استخدام المراحيض العامة أو الحمامات	0.38 (0.79)	0.58
12.	Eating in public	تناول الطعام في الأماكن العامة	0.48 (0.89)	0.53
13.	Taking tests at school	إجراء الاختبارات في المدرسة	0.75 (0.75)	0.54
14.	Talking in a video call	التحدث في مكالمة فيديو	0.39 (0.67)	0.59

Note: M = mean; SD = standard deviation; ρi-it = Spearman’s correlation (discrimination index).

**Table 3:** Models of fit indices.

Model	df; χ^2^	*p*	RMSEA	SRMR	CFI	GFI
1	77; 153.12	0.004	0.068	0.093	0.970	0.986
2	72; 104.67	0.002	0.052	0.089	0.943	0.938

Note: GFI = global fit index; CFI = comparative fit index; SRMR = standardized root mean residual; RMSEA = root mean square error of approximation; df = degree of freedom.

**Table 4:** Spearman’s correlations between the SAPC score and other scales.

	AVCDI	SCAS	CASRS-12	PSA	PHA	SEA	NEA
ρ	0.58	0.53	0.32	0.48	0.43	0.35	0.39
(*p*)	(<0.001)	(<0.001)	(<0.001)	(<0.001)	(<0.001)	(<0.001)	(<0.001)

Note that ρ = Spearman’s correlation.

## Data Availability

The original contributions presented in this study are included in the article. Further inquiries can be directed to the corresponding authors.
